# Supplementary material for: A case–control neuroimaging investigation of chronic Zika virus-infected adults
Source: Front Hum Neurosci. 2026 Feb 20;20:1710905. doi: 10.3389/fnhum.2026.1710905 (PMC12963356; doi:10.3389/fnhum.2026.1710905)
Supplement: Supplementary file 1 [file Table_1.pdf]

Supplementary Materials

Table S1. Neuropsychological assessments

**Groups**	**MoCA**	**Beck Anxiety**	**Beck Depression**	**WHOQOL-BREF**
ZIKV-CNS-GBS	28.42(±2.0)	10.9 (±7.2)	13.9(±7.3)	88.30(±18.16)
nonZIKV-GBS	24.60(±3.2)	14.5(±13.5)	11.2(±9.5)	86.0(±1.07)

Note. MoCA, Montreal Cognitive Assessment; Beck Anxiety, Beck Anxiety Inventory; Beck Depression, Beck Depression Inventory; WHOQOL-BREF, WHO Quality of Life-BREF.
