# Supplementary material for: A case–control neuroimaging investigation of chronic Zika virus-infected adults
Source: Front Hum Neurosci. 2026 Feb 20;20:1710905. doi: 10.3389/fnhum.2026.1710905 (PMC12963356; doi:10.3389/fnhum.2026.1710905)
Supplement: Supplementary file 2 [file Table_2.pdf]

Table S2. White matter lesions identified with LPA-LST

ZIKV-CNS-GBS			nonZIKV-GBS			healthy controls		
Cases	TLV (ml)	TNL	Cases	TLV (ml)	TNL	Cases	TLV (ml)	TNL
# 1	0.659	12	# 1	0.835	16	# 1	0.478	7
# 2	1.012	15	# 2	0.773	19	# 2	0.599	13
# 3	0.919	15	# 3	0.769	10	# 3	0.036	2
# 4	0.268	9	# 4	1.628	20	# 4	5.385	42
# 5	0.643	13	# 5	0.786	12	# 5	0.235	5
# 6	0.879	17	# 6	1.012	22	# 6	0.763	9
# 7	0.466	7	# 7	0.567	10	# 7	1.011	9
# 8	0.621	11	# 8	1.582	12	# 8	0.649	11
# 9	0.661	8	# 9	0.911	16	# 9	2.717	16
# 10	0.211	9	# 10	9.307	48	# 10	0.821	14
# 11	0.088	3	# 11	0.573	9	# 11	0.266	2
# 12	1.122	12	# 12	1.029	16	# 12	0.407	13
# 13	1.061	14	# 13	0.126	5	# 13	0.183	6
			# 14	0.831	14			
			# 15	1.851	18			
N cases	Mean (± SD)	Mean (± SD)	N cases	Mean (± SD)	Mean (± SD)	N cases	Mean (± SD)	Mean (± SD)
N = 13	.66 (± .33)	11.15 (± 3.87)	N = 15	1.55 (± 2.28)	16.50 (± 10.24)	N = 13	1.04 (± 1.47)	11.46 (± 10.20)

Note: LPA-LST, Lesion Prediction Algorithm in Lesion Segmentation Toolbox; N cases, Number of cases with FLAIR imaging data; SD, standard deviation; TLV, total white matter lesion volume; TNL, total number of white matter lesions; m.d.; missing data.
